# A Rapid Review of Available Evidence to Inform Indicators for Routine Monitoring and Evaluation of Respectful Maternity Care

**DOI:** 10.9745/GHSP-D-19-00323

**Published:** 2020-03-30

**Authors:** Patience A. Afulani, Laura Buback, Brienne McNally, Selemani Mbuyita, Mary Mwanyika-Sando, Emily Peca

**Affiliations:** a Institute for Global Health Sciences, University of California San Francisco, San Francisco, CA, USA.; b Independent consultant.; c Africa Academy for Public Health, Dar es Salaam, Tanzania.; d University Research Co., LLC, Chevy Chase, MD, USA.

## Abstract

**Background:**

Some opportunities to routinely capture and improve respectful maternity care (RMC) during facility-based childbirth include quality improvement (QI) initiatives, community-based monitoring efforts through community score cards (CSC), and performance-based financing (PBF) initiatives. But there is limited guidance on which types of RMC indicators are best suited for inclusion in these initiatives. We sought to provide practical evidence-based recommendations on indicators that may be used for routine measurement of RMC in programs.

**Methods:**

We used a rapid review approach, which included (1) reviewing existing documents and publications to extract RMC indicators and identify which have or can be used in facility-based QI, CSCs, and PBF schemes; (2) surveying RMC and maternal health experts to rank indicators, and (3) analyzing survey data to select the most recommended indicators.

**Results:**

We identified 49 indicators spanning several domains of RMC and mistreatment including dignified/nondignified care, verbal and physical abuse, privacy/confidentiality, autonomy/loss of autonomy, supportive care/lack thereof, communication, stigma, discrimination, trust, facility environment/culture, responsiveness, and nonevidence-based care. Based on the analysis of the survey data, we recommend 33 indicators (between 2 and 6 indicators for each RMC domain) that may be suited for incorporation in both facility-based QI and CSC-related monitoring efforts.

**Conclusion:**

Integrating RMC indicators into QI and CSC initiatives, as well as in other maternal and neonatal health programs, could help improve RMC at the facility and community level. More research is needed into whether RMC can be integrated into PBF initiatives. Integration of RMC indicators into programs to improve quality of care and other health system outcomes will facilitate routine monitoring and accountability around experience of care. Measurement and improvement of women's experiences will increase maternal health service utilization and improve quality of care as a means of reducing maternal and neonatal morbidity and mortality.

## Abstract (precis)

We present a set of indicators that could be used to measure the effects of programs on RMC. Integrating these indicators into programs to improve quality of care and other health system outcomes will facilitate routine monitoring and accountability around experience of care.

## BACKGROUND

Documentation of neglectful, disrespectful, and abusive care in health facilities globally has elevated respectful maternity care (RMC) to the forefront of global discussions on the quality of maternity care. Such mistreatment during pregnancy and childbirth care is a violation of human rights. In addition, it deters childbirth in health facilities and may have more direct effects on maternal and neonatal outcomes.^1^^,^^2^

Because addressing disrespect and abuse (D&A) is important to reducing maternal mortality and morbidity, the World Health Organization (WHO) issued a statement calling for greater action, dialogue, research, and advocacy on RMC.^3^ According to the WHO, RMC refers to care organized for and provided to all women in a manner that maintains their dignity, privacy, and confidentiality; ensures freedom from harm and mistreatment; and enables informed choice and continuous support during labor and childbirth.^4^

Early qualitative research on D&A during childbirth in countries such as Ethiopia, Kenya, Mozambique, and Tanzania exposed concerning degrees of D&A,^5^ which precipitated stakeholder demand for taking action to improve women's childbirth experiences. Consequently, there has been much effort to quantitatively measure and describe instances of negative experiences (D&A or mistreatment) as well as positive experiences of maternity care (respectful care), acknowledging that one is not the converse of the other. Much of this work has focused on intrapartum care, although there is emerging evidence that D&A also occurs along the reproductive, maternal, newborn, and child health continuum.^6^^–^^9^

Most quantitative studies have investigated D&A based on the categories proposed by Bowser and Hill: physical abuse, nonconsented care, nonconfidential care, nondignified care, discrimination, abandonment of care, and detention in facilities.^10^ Some have also applied the typologies of mistreatment proposed by Bohren et al (2015) that include these third-order themes: physical, sexual, and verbal abuse; stigma and discrimination; failure to meet professional standards of care; poor rapport between women and providers; and health system conditions and constraints.^5^ However, translation of these concepts into data collection tools has not been consistent. In a review of 5 studies in Ethiopia, Kenya, Nigeria, and Tanzania by Sando et al (2017), the prevalence of D&A ranged from 15% to 98%.^11^ These wide-ranging results in similar contexts highlighted varying methodologies along with various sources of systematic errors related to operational definitions and measurement of the categories, selection of sites and participants, as well as mode, timing, and setting of data collection.^11^

Simultaneously, there has been an increase in work toward development of validated tools that capture women's childbirth experiences. In a systematic review conducted by Nilver et al (review completed in January 2016), they identified 36 instruments developed for measuring various aspects of women's childbirth experiences. Only 7 of the instruments, none of which had been validated in a low- or middle-income country (LMIC), had good psychometric properties.^12^ However, since the review, 2 scales have been developed for measuring women's childbirth experiences in LMICs, both with good psychometric properties—high validity and reliability. These include the RMC perception scale validated in Ethiopia^13^ and the person-centered maternity care scale validated in Ghana, India, and Kenya.^14^^–^^16^ In addition, 2 additional scales have been validated in Canada for measuring respect and autonomy in high-resource settings.^17^^,^^18^ These scales include items that extend the work on women's experiences beyond measuring D&A to measuring various positive aspects of women's childbirth experiences. The questions in these scales, as well as questions in various prior questionnaires for surveys and birth observations on women's experiences, provide useful indicators for evaluating interventions to improve women's childbirth experiences.

Further, WHO's Network for Improving Quality of Care is working with 10 pilot countries to incorporate a modest set of common quality of care indicators for routine monitoring of maternal and newborn care. The Network's monitoring framework includes a flexible set of indicators for each WHO quality of care domain, including the 3 experience of care domains. The small set of common measures includes a mix of health outcome, processes of clinical care, infrastructure/input indicators, and 3 initial RMC indicators, namely: proportion of women who receive predischarge counseling, experience verbal or physical abuse, and who are able to have a companion of choice.^19^ Other process and outcome indicators related to RMC are detailed in the WHO standards for quality of maternal and neonatal health care.^20^ Although these are positive steps, how to incorporate indicators and support sustained measurement of women's experiences of care from their perspective remains a challenge.

There is an opportunity to translate the lessons learned from the early and largely well-funded research studies on RMC into feasible and sustainable routine indicators of experiences of care. Opportunities to routinely capture experience of care include facility-based quality improvement (QI) initiatives, community-based monitoring efforts through accountability mechanisms such as community score cards (CSC), as well as performance-based financing (PBF) initiatives. QI interventions vary in form but generally consist of a structured approach to measure and improve quality of care on a continuous basis. Their objective is to improve quality based on the findings of routine quality assessments, which could be through surveys, observations, or other methods.^21^^–^^23^ CSCs and similar approaches like citizen report cards have a similar aim of continuous improvement but through accountability mechanisms led by the community.^24^ They seek input from users on experience of care, often using participatory methods such as community meetings or consultations to get feedback from both users and providers to guide improvement efforts.^25^^–^^27^ PBF, on the other hand, is generally a mechanism to measure and incentivize providers' and facilities' performance: facilities and providers earn incentives based on achievement of specific performance criteria.^21^^,^^28^^,^^29^

For this review, we wanted to consider widely used approaches and platforms with potential to generate routine data for accountability on RMC in lower-resource settings. QI or assurance approaches have been used in LMICs for more than 30 years and are a major source of investment by major donors such as the United States Agency for International Development (USAID). The modern paradigm of improving health care quality has been characterized as quality management with a “focus on the client, systems and processes, teamwork, and the use of data.”^30^ There are examples of QI as a tool to advance RMC with the potential to learn more about how to sustain and institutionalize these opportunities to use data on experiences of care for QI.^31^^,^^32^

CARE's CSC social accountability approach (and other similar models) can contribute to improvements in service availability, access, utilization, and quality.^33^ CSCs allow for systematic collection of feedback (or data) to improve public services and have been used to inform dimensions of quality such as user-centered indicators like providing respectful care, listening to patients, and respecting privacy.^24^^,^^33^

PBF programs have become a popular development approach for improving quality of care. A recent review identified 32 programs in 28 LMICs, which collectively produced a total of 68 quality measurement tools.^28^ Maternal and child health was often a focus and granting of rewards were based on measuring performance. Although the incentives were largely tied to aspects of structural quality versus process and outcome measures, there is potential to include RMC measures (now conceptualized as important aspects of quality) within these schemes.

Given more recent efforts and potential to advance RMC through PBF, QI, and CSC, these platforms were chosen as a starting point for investigation into identifying D&A/RMC indicators for monitoring, accountability, and improvement. This review addresses the dilemma of deciding which D&A/RMC indicators may be best suited for routine monitoring. Integrating such indicators into existing operational schemes for QI may provide stakeholders with a solution to the ongoing challenge of routine RMC improvement.

Integrating RMC indicators for routine monitoring into existing QI operational schemes may provide stakeholders with a solution to the challenge of routine RMC improvement.

The objective of this rapid review was to provide practical evidence-based recommendations on indicators that are best suited for more routine measurement of RMC and D&A, using QI, CSCs, and PBF initiatives as example platforms. This article is not endorsing or promoting the aforementioned (or any other) approaches to addressing D&A/RMC. Instead, we are recommending indicators that can be integrated into existing platforms.

The review was originally motivated by the authors' engagement in the Global Respectful Maternity Care Council^34^ and inspired by the USAID-funded Health Evaluation and Applied Research Development (HEARD) project's ^35^ activities in East Africa, during which national decision makers posed the question: “what indicators should we use for monitoring performance on RMC?” The answer is not simple, and the work required to best answer the question can easily result in the general status quo: exclusion of RMC indicators from routine measurement. Therefore, to advance the thinking around which indicators are most suitable, this article presents available existing indicators to inform selection and testing of indicators in LMIC contexts and can serve as a useful starting point for future consultations to support decision making.

We present available existing indicators to inform selection and testing of indicators in LMIC contexts.

## METHODOLOGY

Guided by the goal of this project, which was to provide timely evidence to make recommendations for the inclusion of RMC indicators for routine monitoring, we used a rapid review approach. Rapid review has been defined as “a type of knowledge synthesis in which systematic review processes are accelerated and methods are streamlined to complete the review more quickly than is the case for typical systematic reviews.^36^ Systematic reviews take at least a year to complete, but rapid reviews take an average of 5–12 weeks to complete. Therefore, they are able to provide evidence within a shorter time frame to inform health policy, programming, and systems decisions.^36^^,^^37^

The rapid review had 3 phases. We first started with a review of handpicked existing documents to identify indicators that have been used to measure RMC or D&A/mistreatment. These initial documents included the RMC indicator compendium by the RMC measurement workgroup of USAID's Maternal and Child Health Integrated Program and the WHO standards for improving quality of maternal and newborn care in health facilities, which includes standards related to RMC.^20^^,^^38^^,^^39^ We also reviewed all the quantitative studies in the mixed-methods systematic review by Bohren et al that are also described by Sando et al in an article on methods used in prevalence studies for D&A.^5^^,^^11^^,^^40^^–^^45^ In addition, we included 2 articles describing the validation of scales for measuring women's experiences in LMIC settings.^13^^,^^14^

From these documents and publications, we identified indicators of RMC and D&A/mistreatment and extracted them into a Microsoft Excel spreadsheet by: domain, indicator, source of indicator, specific questions asked or observation instructions, type of indicators (observed or self-reporting), how the indicator was used (e.g., exit survey, facility-based routine data collection, etc.), where the indicator was used (country and population), if it was validated, and full citations of documents/papers in which the indicator was referenced. We then updated the spreadsheet by searching on the Internet for additional publications on RMC and D&A. We reviewed the RMC council resources page and searched on PubMed using the key words “respectful maternity care,” “mistreatment,” “disrespect and abuse,” and “person-centered maternity care” published between 2015 and the time of the review (October 2017). We focused on indicators that could be measured quantitatively. In total, we reviewed 35 articles on D&A and RMC.

The second phase of the rapid review identified RMC/D&A indicators that had been used in CSCs using the keywords “maternal health,” “obstetrics,” “respectful maternal care,” “labor and delivery,” and “community score cards” in PubMed and Google Scholar searches. Five articles were identified from this review, and the indicators from these documents were extracted into the spreadsheet. Since few indicators had actually been used in the CSCs, we also included RMC indicators mentioned as potentially useful in CSCs. In addition, we reviewed 11 handpicked articles to identify RMC indicators that could be used for PBF. We then updated the indicator spreadsheet to identify which could potentially be used in CSCs and PBF schemes. All papers reviewed are shown in Supplement 1.

Finally, for the third phase of the rapid review, we developed a questionnaire with the list of indicators to survey the RMC and other maternal and child health experts. We asked respondents to select which of the indicators they would recommend for use in QI, CSCs, and PBF initiatives, whether they had used any of the indicators in their work, if they have been involved in developing measures for any of the indicators, if they had any concerns about any of the indicators, and if there were any indicators that they were aware of that were not included in the list provided. In addition, we collected demographic data on participants including gender, age, years of work experience in maternal and child health, continents and countries where they currently worked, type of organization in which they worked, main area of work, and whether or not they had been involved in PBF or CSC projects. The survey was in English and self-administered online. It was conducted using the RedCap application^46^ and distributed to the RMC council and Health Information and Publications Network listservs, as well as distributed directly to individuals involved in RMC measurement.

After conducting the survey, we analyzed the data and ranked the indicators based on their frequency of selection. We then grouped the indicators by the top 3 selected for each RMC domain for QI, CSC, PBF. The domains were based on classifications used in the various studies reviewed, which included a combination of domains from various prior frameworks including Bowser and Hill's classification, the typologies of mistreatment, and WHO's quality of care framework. We organized the domains to ensure they captured all domains previously used but avoided as much overlap as possible (recognizing that most RMC domains are not mutually exclusive). Next, we reviewed each of the top 3 indicators in each domain to assess their importance, feasibility of measurement, if they included or were missing key indicators, and if there were any particular concerns raised about them. We reviewed the qualitative data from the responses to the open-ended questions for additional feedback on the indicators from the survey to guide this process.

## RESULTS

From the rapid review we identified 49 indicators spanning several domains of RMC and mistreatment including dignified/nondignified care, verbal and physical abuse, privacy/confidentiality, autonomy/loss of autonomy, supportive care/lack thereof, communication, stigma, discrimination, trust, facility environment/culture, responsiveness, and nonevidence-based care. These indicators had been used as part of questionnaires and self-reported in exit interviews or in community surveys or as part of checklists for direct observations during labor and delivery and broader facility assessments. The full set of indicators extracted from the review are shown in Supplement 1 with details on their sources and if they had been used in QI, CSC, or PBF initiatives.

Thirty-seven people responded to the survey (34 female). Respondents worked in various regions around the world: 16 in East Africa; 6 each in West Africa, Southern Africa, Asia, and North America; 5 in Europe; and 4 in South America. Eighteen worked in academic institutions, 12 in government institutions, 10 in nonprofit institutions, and 2 in for-profit institutions. Most (25) were engaged in research, with between 3 and 8 in other activities such as teaching, clinical practice, program management and implementation, advocacy, and other related fields. Thirty respondents had never been involved in CSC or PBF initiatives. The indicators in the survey (Supplement 2) are ranked by how frequently the respondents selected them for QI, CSC, and PBF.

Almost all the indicators (about 46 of the 49 indicators) were selected by more than half of respondents for QI and CSC. However, only 12 of the indicators were selected by more than half of the respondents for PBF. In addition, there was concern among some respondents that it was premature to recommend specific indicators for PBF programs without testing them and also that RMC-related behaviors should be normative and not rewarded. Thus, we decided to present only indicators for QI and CSC as more work needs to be done to be able to recommend RMC indicators for PBF. This process led to a set of 33 indicators for QI and CSC, which includes between 2 and 6 indicators for each RMC domain that could be used for both facility-based QI and CSC initiatives (Table). All of the indicators except women's perception of wait time and trust could be obtained from both surveys and direct observations.^13^^,^^14^^,^^39^^–^^45^

**Table.** Potential Respectful Maternity Care Indicators for Quality Improvement and Community Score Cards, by Domain

	Potential Information Source	
	Community-Based/Exit Surveys With Women	Facility Assessments/Observations
Dignified care		
1. Women treated with respect (subject to women's/local interpretation)	X^a^	X
2. Providers introduce themselves to women	X	X
3. Women treated in a friendly manner (subject to women's/local interpretation)	X	X
4. Women called by name	X	X
Privacy and confidentiality		
5. Physical privacy ensured (e.g., examined behind screens or curtains and other physical visual barriers)	X	X
6. Auditory privacy ensured (Private patient health information not heard by others)	X	X
7. Patient records and medical files are kept confidential (not accessible to people not involved in care provision)	X	X
No abuse		
8. No verbal abuse (insults, intimidation, shouting, scolding, threatening)	X	X
9. No physical abuse (slapping, hitting, pushing, pinching, restraining, or otherwise beating the patient)	X	X
10. No episiotomy given or sutured without anesthesia	X	X
Autonomy		
11. Providers explain to women what to expect and any medications administered, or procedures performed	X	X
12. Women give informed consent prior to procedures and examinations	X	X
13. Women and family involved in care (e.g., decision making on treatment and procedures)	X	X
14. Women allowed to assume position of choice during labor and delivery	X	X
Communication		
15. Women encouraged to and able to ask questions	X	X
16. Providers speaks to women in a language and at a language-level that they understand	X	X
Supportive care		
17. Women allowed to have choice of companion during labor and delivery	X	X
18. Not denying women care (e.g., refusing care for any reason)	X	X
19. Not abandoning women during labor and delivery (e.g., not responding to woman's call for help)	X	X
20. Providers ask about emotional feelings and concerns of women	X	X
21. Women trust staff (subject to women's interpretation)	X	—
Facility environment		
22. Cleanliness of facility	X	X
23. Facility is perceived safe	X	X
24. Facility not overcrowded/woman has own bed	X	X
25. Facility has electricity	X	X
26. Facility has water	X	X
27. Enough providers		
Responsiveness		
28. Perception of wait time	X	—
29. Actual wait times	X	X
30. Payment/equity/cost		
31. No discrimination or poor treatment based on ethnicity, race, economic status, HIV status, birth outcomes, age, number of children	X	X
32. Not requesting bribes or informal payments	X	X
33. Women not detained at facilities due to lack of payment	X	X
34. Health care services affordable for all	X	X

a X denotes that it can be obtained from relevant potential data source.

Supplement 1 has full list of sources for each indicator.

## DISCUSSION

We have shared a set of indicators spanning various domains of RMC that might be used to measure program impacts on RMC. Although the initial focus of the review was for QI and CSC initiatives, these indicators can be used in other programs to improve women's experiences including for routine monitoring of RMC indicators in comprehensive maternal and neonatal programs or as part of other programmatic approaches to reduce mistreatment and improve RMC. The recommended indicators are a useful pool to draw from. However, selection of indicators for any setting should be preceded by a local review process by local experts and key stakeholders at the appropriate level (e.g., unit and facility leaders, community leaders, national policy and planning officials, bureaus of statistics, health management and information system designers, and managers, depending on the level of the initiative).

These indicators can be used for QI and CSC initiatives and in other programs to improve women's experiences.

The goal of this process was to create a parsimonious list of measures that are core to RMC or respectful care more generally that could be feasibly measured. Thus, the list is neither exhaustive nor meant to be prescriptive. For users who prefer a more extensive list of indicators, Supplement 1 is a useful reference. Also, this process focused on identifying the indicators that have been or could be used rather than how to measure them. The indicators in the Table include some that have been used as part of validated scales that have undergone psychometric analysis and others that have been used in surveys and observations without a formal validation process. For those that are part of validated scales, the scales could serve as important measurement tools to collect data on the indicators. For those that are not part of validated tools, they could still be measured as standalone questions in surveys or observations, with careful attention to wording of survey questions and observation prompts.

So, how do decision makers arrive at how many indicators and which ones? How the indicators are selected and used depends on local policy and program goals. Key questions to consider are: what are the prioritized aspects of respectful care in the context; whose perspectives do you want to measure and what are the feasible method(s) by which to measure these perspectives; and how can these indicators fit into existing data collection systems? These questions address implementation science goals of engaging stakeholders and marshalling the best evidence in support of decision makers with the goal of improving implementation outcomes such as acceptability, adoption, appropriateness, feasibility, fidelity, implementation cost, penetration, and sustainability of efforts related to RMC.^47^^,^^48^ Applicability of the indicators may differ for locally driven compared to globally driven programs, and these need to be considered in the selection of indicators.

Decision makers can choose the indicators to include based on local policy and program goals.

Other considerations include whether programs are interested in capturing more than discreet instances of D&A. To assess women's experiences more broadly on a continuous scale, we recommend using validated scales such as the person-centered maternity care^14^^–^^16^ and RMC perception scales.^13^ These scales can be used to generate experience of care scores that rate women's experiences of care from their perspective. Other programs might be interested in just the proportion of women who experienced certain aspects of care based on their intervention elements or targeted priority areas. For example, the proportion of women who were allowed a companion during labor and/or delivery, the proportion allowed to choose their birth position, the proportion verbally or physically abused, and proportion who experienced discrimination. Such needs could be met by including specific individual items in surveys or observation checklists. The scales could also be used to obtain such individual percentages as well as scores in both prevalence studies and program evaluation.^16^^,^^49^

Respectful care is a complex and multidimensional construct that is not well-captured by a single satisfaction measure in which responses are often inflated and lack sufficient specificity to be actionable.^7^^,^^50^^,^^51^ Also, just because a woman was not verbally or physically abused does not mean she was treated with dignity and respect. Additionally, experiences of respect may be intermixed with disrespect. For example, a woman could have been allowed a birth companion and still have received very little information or consent related to her care. Further, we want to avoid the idea that the implementation of one (albeit important) action, like hanging curtains for privacy, means respectful care was achieved. Therefore, we suggest a careful consideration of the domains of mistreatment and respectful care and generally recommend multiple indicators for a more wholistic approach to assessing client experiences. Each domain in Table includes 2–6 indicators, and we recommend selecting at least 1 to 2 indicators from each domain based on program goals.

Respectful care is a complex construct that is not well-captured by a single satisfaction measure in which responses are often inflated and lack sufficient specificity to be actionable.

Irrespective of which indicator is selected, careful attention is needed for using it in a particular context. The person-centered maternity care scale items capture most (but not all) of the indicators and includes items in all domains except payment/equity/cost.^16^ Therefore, it is a useful resource for measuring the indicators in QI, CSC, or other types of RMC interventions.^49^ For the indicators not included in the scale, reviewing how those indicators have been used in prior studies and then testing in the setting will ensure questions are relevant, understandable, and measure what is intended.

Each program will also need to decide the most feasible and useful way to obtain information in their setting. The recommended indicators can be integrated into exit interviews with women, community-based surveys, as well as facility assessments/observations. However, each method has its limitations. Exit surveys are the most easily conducted but also have the highest bias from social desirability (i.e., women responding based on what they think is acceptable to say rather than based on what they actually experienced). For example, women may not want to report negative experiences when interviewed at the facility due to fear of retaliation. Observations may be the most objective, but they are also the most labor and time intensive, and observers' reports may not necessarily reflect women's experiences. Community surveys may be the best approach for CSCs since they likely will involve community participation or better still may be community led. Community participation in the overall project will facilitate entry into the community for data collection. The most cost-effective approach will depend on existing data collection mechanisms in the particular setting. Where there is no existing data collection mechanism to integrate RMC indicators, facility-based exit surveys of women who have recently given birth might be the least expensive approach. However, the limitations of the approach selected must always be kept in mind.

In each case, quality training of all data collectors is essential, regardless of previous QI or CSC experience, due to the complex, sensitive, and subjective nature of many RMC indicators. Other key considerations will be related to sampling and timing of interviews or observations. In addition, integrating these indicators in routine health systems is challenging due to their need of extra workforce to collect the data and thus the need to advocate with policy makers to integrate them into health information systems.

Although this review focused on quantitative indicators, it is important to recognize the important role of qualitative data for more nuanced understanding of women's experiences of care. A mix of quantitative and qualitative methods of data collection and analysis are most optimal for understanding and monitoring women's experiences of childbirth care to inform program implementation. The development of local expertise in mixed methods will help support routine monitoring of quantitative indicators as well as the periodic application of qualitative methods (e.g., in-depth interviews or focus groups on a quarterly/semi-annual basis).

### Limitations and Strengths

A potential limitation of this process was that we used a rapid review approach, which is less rigorous than a systematic review. However, rapid reviews are useful for synthesizing information in a more timely manner. In addition, given that the team involved in this work had prior experience in measurement and there were recent documents and reviews of RMC indicators, a rapid review was an efficient approach to promptly recommend RMC indicators. Rapid reviews are specifically useful for new and emerging research topics, as well as for assessing the amount of information available, which applies to a project for assessing the applicability of RMC indicators in other initiatives. The expert review component complemented the rapid review to increase the robustness of the recommended indicators. Expert reviews are often used in the user experience domain of the technology industry for new products to provide fast, practical input based on the usability of a potential product. The principle is that experts know the domain very well, in this case RMC and maternal and newborn health, so they can provide recommendations for usability and feasibility.

Another limitation was the fact that we focused on only indicators for QI, CSCs, and PBF initiatives and were not able to recommend indicators for PBF. There is potential to include RMC indicators in PBF initiatives, but this requires further research. Additionally, we focused on indicators mostly used in the intrapartum period, even though RMC is important beyond the intrapartum period. For example, a growing body of research has highlighted that women also have poor experiences during prenatal care.^6^^–^^9^ Thus, there is a need for indicators to track women's experiences along the reproductive, maternal, newborn, and child health continuum. Some of the indicators recommended have been used to measure women's experiences during prenatal care,^9^ but more work is needed in this area. Furthermore, we did not include indicators that capture the provider experience and structural/systems-level drivers of mistreatment. These are important but require more work to make evidence-based recommendations.

The indicators each have their own limitations, depending on the implementation, context, and method of data collection. Some indicators are more objective (relatively), such as whether a provider introduces himself, if physical privacy is observed, or if there is verbal or physical abuse. However, some indicators such as being treated with respect and involving women in care, or trust, are more subjective and context-specific—whether self-reported or externally observed. Although factors related to respectful care such as infrastructure (e.g., supplies, infrastructure) are easiest to capture, real system improvements will only be informed through the inclusion of self-report from the client and provider perspectives. Social desirability and recall bias are limitations where indicators are self-reported, and the extent varies depending on place and timing of interviews. Observations may be more objective but subject to Hawthorne effect—providers may perform better than normal when being observed—and not directly capture women's experiences. The Hawthorne effect has not been a big issue so far in some studies as providers may not identify their behavior as mistreatment.^31^^,^^52^^,^^53^ But as provider awareness of RMC increases, it may become a bigger issue during observations. However, we will continue to grapple with these issues in any work involving assessing people's experiences and decisions will have to be made carefully to balance relevance, accuracy, and feasibility.

Since we completed the review, there have been other quantitative studies measuring RMC. We did not attempt to systematically update our indicators. However, most of these studies measure mistreatment based on previously used indicators that were captured in the review. A key addition to the literature are the WHO tools for measuring how women are treated during facility-based childbirth that focus on measuring mistreatment through community surveys and labor observations.^54^ However, the focus is on measuring existing indicators, most of which are captured in our original list. Notwithstanding, we note that the recommended indicators are intended to be comprehensive and representative of key domains of RMC but not exhaustive. Thus, we recommend these indicators as a guide and starting point for a consultative process to identify relevant RMC indicators for programs.

## CONCLUSIONS

Although this review was initially motivated by our collaborative activities in East Africa and burgeoning efforts to use QI and CSC to advance RMC, the indicators could be used more globally by program implementers with objectives to measure and improve client experiences of care. Early implementers of these indicators are encouraged to document their experiences and lessons learned, particularly as they relate to the incorporation of indicators into routine monitoring and evaluation systems. Methodological work on RMC measurement is growing, and additional indicators may evolve from this process. QI and CSC initiatives are just 2 ways of improving RMC at the facility and community level through accountability mechanisms. Additionally, the opportunity for integration of RMC in PBF initiatives needs further exploration and research. There may also be other quality and performance improvement efforts that can adopt RMC indicators.

We hope that feasible, sustainable efforts to institutionalize monitoring and evaluation of D&A/RMC by local institutions emerge from this analysis—supported by continued partnership among all actors involved in these endeavors. Higher-level advocacy is needed for policies to assure RMC across all levels of the health system, and community-level interventions are needed to empower women and the community at large on their rights and knowledge of RMC. Such interventions will facilitate global efforts to increase maternal health service utilization and improve quality of care as a means of reducing maternal and neonatal morbidity and mortality.

## Supplementary Material

19-00323-Afulani-Supplement1.xlsx

19-00323-Afulani-Supplement2.xlsx

## Notes

**Peer Reviewed**

**First published online:** March 19, 2020

**Funding:** This study was funded by the United States Agency for International Development (USAID) under the Health Evaluation and Applied Research Development (HEARD), Cooperative Agreement No. AID-OAA-A-17-00002. This study is made possible by the support of the American People through USAID. The findings of this study are the sole responsibility of University Research Co., LLC and do not necessarily reflect the views of USAID or the United States Government.
